# The effect of nurses' perceived workplace incivility on their presenteeism and turnover intention: The mediating role of work stress and psychological resilience

**DOI:** 10.1111/inr.12950

**Published:** 2024-03-11

**Authors:** Ayhan Durmuş, Özgün Ünal, Halil Türktemiz, Yunus Emre Öztürk

**Affiliations:** ^1^ Faculty of Economics and Administrative Sciences, Department of Health Administration Yozgat Bozok University Yozgat Turkey; ^2^ Sakarya Business School, Department of Health Administration Sakarya University Sakarya Turkey; ^3^ UNEC Research Center of Health Economics Azerbaijan State University of Economics (UNEC) Baku Azerbaijan; ^4^ Vocational School of Health Services, Medical Imaging Techniques KTO Karatay University Konya Turkey; ^5^ Faculty of Health Sciences, Department of Health Administration Selcuk University Konya Turkey

**Keywords:** Nursing management, presenteeism, psychological resilience, turnover intention, work stress, workplace incivility

## Abstract

**Aim:**

This study aims to determine the effects of nurses' perceived workplace incivility on nurses' presenteeism and turnover intention and to reveal the mediating role of work stress and psychological resilience in the possible impact.

**Background:**

Nurses directly contribute to the treatment of patients. The problems nurses encounter in the workplace can negatively affect nurses' attitudes towards work. Therefore, the problems faced by nurses should be determined.

**Methods:**

This study complies with the STROBE checklist. This cross‐sectional survey was conducted with 302 nurses working in a university hospital in the Konya province of Turkey. Data were collected in May–July 2021. The questionnaire consisted of six parts: sociodemographic characteristics form, workplace incivility scale, psychological resilience scale, work stress scale, turnover intention scale and presenteeism scale. The data were analysed using descriptive statistical methods and partial least‐squares path analysis.

**Results:**

It was determined that workplace incivility positively and significantly affected turnover intention, presenteeism and work stress. In contrast, it negatively and significantly affected psychological resilience. In addition, psychological resilience played a mediating role in the effect of workplace incivility on presenteeism.

**Conclusion:**

The results reveal that the behaviours of incivility encountered by nurses in the workplace increase their presenteeism and turnover intention, and work stress further strengthens these effects. The psychological resilience of nurses is a factor that can help them eliminate their negative emotions and attitudes. Therefore, it is recommended that nursing and health managers first identify the stress factors in the workplace and be determined to fight them. In addition, organizing training and providing psychological support to increase nurses' psychological resilience may enable nurses to develop more positive feelings about their jobs and workplaces.

**Implications for nursing and health policy:**

Nursing and health managers must determine workplace incivility behaviours and inform all employees about these behaviours, their consequences and how to deal with such incivility. In addition, nursing and health managers must determine the stress factors in the workplace and be adamant about combating these factors. In addition, nursing and health managers must give importance to training that will increase the psychological resilience of nurses.

## INTRODUCTION

The essence of nursing is centred on ‘care’, manifested through various actions aimed at treating or alleviating ailments caused by a disease process and promoting and maintaining overall health (López‐Verdugo et al., 2021). Nurses are among the health workers most interested in the patient while providing health services, and they are vital in all healthcare settings.

Negative behaviours observed in health institutions are exhibited by patients, patients’ relatives, colleagues or any other persons and consist of threats, insults, physical harm or sexual assault that create security problems for the employee health (Aydın, 2004). Aggressive behaviours negatively affect organizational peace, communication, individual and organizational productivity, effectiveness and performance (Aydıntan & Göksel, 2010). Workplace incivility, which has become a harmful behaviour with significant adverse organizational and individual effects, especially in recent years, is one of these undesirable behaviours (Kumral & Çetin, 2016).

Workplace incivility is defined as low‐intensity behaviours that violate respect and workplace norms and are performed to harm the target (Spence Laschinger et al., 2009). Such behaviours are uncivilized, rude and disrespectful (Reio & Ghosh, 2009). Workplace incivility starts with rude behaviour and turns into harmful acts called bullying, sexual harassment or workplace violence (Phillips et al., 2018). Nurses can encounter diverse challenges, including incivility, bullying and workplace violence, which harm the quality of healthcare services (Aljuaid & Alharbi, 2023). When nurses face workplace incivility, it can negatively affect nursing practices, patient care and general health (Ricciotti, 2016). In addition, workplace incivility increases nurses' turnover intention, work stress and absenteeism; decreases job satisfaction and productivity (Taşkaya & Aksoy, 2021; Taştan, 2014). To not decrease the quality of patient care, it is necessary to determine the negativities in the nurses' working environment and take the necessary precautions.

The reasons for leaving the job are unfavourable working conditions, unmet job expectations and personal factors (Pin‐Pin Choi et al., 2012). Even if the turnover intention does not result in job quitting, it has crucial effects on the organization (Chao et al., 2015). Employees' turnover intention will cause their performance to decrease and lead to various damage to the institution (Erkuş & Afacan Fındıklı, 2013).

Workplace incivility damages the social identity in the workplace, disrupts the harmony between employees and organizational norms and weakens the emotional bonds of employees (Leiter et al., 2015). In such a case, the individual tends to go to work reluctantly due to the possible harm that may occur; that is, he exhibits presenteeism behaviour (Ayrancı & Kumral, 2020). Thus, employees continue to work unwillingly and inefficiently (Conway et al., 2016). Presenteeism is defined as ‘Individuals' not doing or being unable to do their work efficiently, even though they are physically present at the workplace, for various reasons such as illness, mental disorder, or unwillingness to work’ (Ruhle et al., 2020). Nursing is among the professions with high presenteeism in health institutions, negatively affecting work quality (Simonetti et al., 2021).

Nurses are tending to stress, anxiety, panic attacks, depression, sleep disorders and loss of self‐esteem due to the effects of workplace incivility (Taşkaya & Aksoy, 2021). Stress is a mental, physiological and physical condition that occurs when a person faces an environment that is challenging to adapt to (Li et al., 2018). The work stress experienced by nurses can adversely affect their physical and psychological well‐being (Faremi et al., 2019). In such a situation, the psychological resilience of nurses gains importance. Psychological resilience is defined as the ability of an individual to cope with negativity, uncertainty, conflict, failure and many similar negative situations and consequently to be successful (Luthans, 2002). It is stated that the psychological resilience of individuals is an essential factor in coping with stress (Oginska‐Bulik & Michalska, 2021). Stress increases the individual's resilience by teaching them to overcome stress (McManama O'Brien et al., 2021). Although some individuals surrender to psychological disorders such as anxiety in stressful and traumatic events, others can quickly get rid of the negative mental state and continue their daily lives (Bozdag & Ergün, 2021). It can be stated that individuals who can get rid of the negative situations they encounter in a short time have high levels of psychological resilience. Psychologically sound individuals will be advantageous in coping with the negativities they encounter in the workplace and in fulfilling new responsibilities (Karaman et al., 2020). Therefore, resilience can protect employees from the negative consequences of work stress (Oginska‐Bulik & Michalska, 2021).

### The objective and hypotheses of the research

There is a serious shortage of health workers in Turkey. When OECD data are examined, the rate of nurses per 1,000 people in Türkiye is 2.4 (OECD, 2021), far below the OECD average (8.8‰). This situation can bring unfavourable working conditions. An insufficient number of employees in services that need to be maintained uninterruptedly, such as health services, may cause employees to assume additional roles outside their duties (Okyere, 2018). Because the number of health workers is insufficient in Turkey, the great importance attached to employee satisfaction by researchers, policymakers, and managers makes it necessary for managers to understand the factors that significantly affect the performance of their organizations (Kuzey, 2012). For this reason, it is extremely important to perceive the changes that may occur in the attitudes and behaviours of the employees due to the lack of employees.

The literature shows that nurses' work incivility affects their turnover intention and presenteeism (Arslan Mehmood et al., 2023; Taylor et al., 2021). In addition, different researchers have stated that job stress is associated with turnover intention and presenteeism (Jiang et al., 2021; Mehmood et al., 2023). For this reason, work stress may have a mediating role in the effect of incivility that nurses are exposed to, turnover intention and presenteeism. Psychological resilience can be an important factor in protecting the mental health of healthcare professionals and reducing their stress levels (Deniz & Ünal, 2023). In this context, it is thought that the psychological resilience of nurses may have a mediating role in coping with the negative effects of workplace incivility.

In light of the information in the relevant literature, it is possible to state that the uncivil behaviours that nurses encounter in the workplace can affect nurses negatively in many ways. In this context, this study aims to determine the effect of incivility that nurses encounter in the workplace on the nurses' presenteeism and turnover intention and to reveal how work stress and psychological resilience mediate the possible effect. Hypotheses have been developed in line with the purpose and conceptual framework of the study.

*H_1_: Workplace incivility affects psychological resilience*.

*H_2_: Workplace incivility affects work stress*.

*H_3_: Workplace incivility affects turnover intention*.

*H_4_: Workplace incivility affects presenteeism*.

*H_5_: Psychological resilience affects turnover intention*.

*H_6_: Psychological resilience affects presenteeism*.

*H_7_: Work stress affects turnover intention*.

*H_8_: Work stress affects presenteeism*.

*H_9_: Psychological resilience has a mediating role in the effect of workplace incivility on turnover intention*.

*H_10_: Psychological resilience has a mediating role in the effect of workplace incivility on presenteeism*.

*H_11_: Work stress has a mediating role in the effect of workplace incivility on turnover intention*.

*H_12_: Work stress has a mediating role in the effect of workplace incivility on presenteeism*.

## METHOD

### Design and sample

A descriptive correlational cross‐sectional survey design was used for this study. The Strengthening the Reporting of Observational Studies in Epidemiology (STROBE) guidelines were used to report this study.

The study population comprised approximately 750 nurses working in a university hospital in Konya, Turkey. Nurses usually worked in rotation at the hospital, where the day shift started at 8 am and ended at 6 pm, and the night shift started at 6 pm and lasted until 8 am. Nurses generally worked consecutively day and night shifts. The convenience sampling method was used to select the study's sample. Participants were informed about the study, and those who volunteered were included. Only one response per person is allowed. Within the scope of the study, 302 nurses were reached who agreed to participate voluntarily. Inclusion criteria were nurses who worked at the hospital for a minimum of one year and worked during the COVID‐19 pandemic. Exclusion criteria were working in leadership positions or not providing direct patient care.

### Instruments

The data were collected using the ‘Sociodemographic characteristics form’, ‘Workplace Incivility Scale (WI)’, ‘Psychological Resilience Scale (PR)’, ‘Work Stress Scale (WS)’, ‘Turnover Intention Scale (IQ)’ and ‘The Stanford Presenteeism Scale (P)’.

### Sociodemographic characteristics form

The sociodemographic characteristics form consists of five questions about participants’ gender, age, educational status, professional experience and personality. The self personality evaluation of the participants was measured with the ‘How would you describe your personality?’ question. As an answer to the question, the participants were asked to choose one of the most appropriate ‘anxious’ or ‘relaxed’ options.

#### Workplace incivility scale

The scale developed by Cortina et al. (2001) and Gök et al. (2019) conducted the Turkish validity study. The scale consists of seven statements. Participants were asked whether they had been exposed to rude behaviour by their managers or friends in the last year at their current institution. The scale includes sample items such as ‘put you down or condescending to you’ and ‘made demeaning or derogatory remarks about you’. The statements of scale are encoded as 1 (never) and 5 (quite often). The Cronbach's alpha value in the current validation study of the scale was 0.92.

#### Psychological resilience scale

Smith et al.’s (2008) psychological resilience scale was used to measure the psychological resilience of participants. Doğan (2015) conducted the scale's Turkish validity and reliability studies. The scale consists of six statements. Items 2, 4 and 6 in the scale are reverse coded. High scores obtained after reverse‐coded items were translated indicate a high level of psychological resilience. Example items are ‘I can recover quickly after difficult times’ and ‘I have difficulty overcoming stressful events (R)’. The statements in the scale are encoded as 1 (strongly disagree) and 5 (strongly agree). The Cronbach's alpha value in the scale validation study was 0.83.

#### Work stress scale

The scale was developed by Rizzo et al. (1970), and the Turkish validity study conducted by Gül (2007) was used to measure work stress. The scale consists of five statements. The statements in the scale are encoded as 1 (strongly disagree) and 5 (strongly agree). The Cronbach's alpha value in the validation study of the scale was 0.91.

#### Turnover intention scale

The scale was developed by Camman et al. (1979), and the Turkish validity study conducted by Akbolat et al. (2021) was used to measure turnover intention. The scale consists of statements about the participants’ desire to leave the hospital where they currently work and work in another institution. The high average of scale indicates the intention of participants to leave their institutions if they find any opportunities. The scale consists of three statements (e.g., I often think of leaving the hospital where I work). The statements in the scale are encoded as 1 (strongly disagree) and 5 (strongly agree). The Cronbach's alpha value in the validation study of the scale was 0.80.

#### The Stanford presenteeism scale (SPS‐6) (P)

The scale was developed by Koopman et al. (2002), and Turkish validity and reliability studies were conducted by Moç (2018). The Stanford Presenteeism Scale (SPS‐6), which consists of six statements (e.g., It is very difficult to deal with stressful situations related to my job), is encoded as 1 (strongly disagree) and 5 (strongly agree). The Cronbach's alpha value in the validation study of the scale was 0.89.

### Data collection process

The data were collected between 1st May and 31st July 2021, utilizing face‐to‐face interviews and online survey. The online survey was used as an alternative method since the nurses had a high workload and did not want to spend their rest time filling out the questionnaire while working. Data collection tools were transformed into a Google Survey which was sent to participants online via WhatsApp messages or email. Participants were asked to click the Google Surveys link in the message and complete the forms. A survey link was sent to the participants twice a week throughout the research to remind them to complete the forms. Also, the researchers collected data through face‐to‐face interviews. Participants were ensured to be alone so they did not feel pressured while completing the survey. The average time to fill out the form was 10 minutes, and no incentives were given to the participants.

### Data analysis

Descriptive statistical methods were used in the data analysis, whereas partial least squares path analysis (PLS‐SEM) was used in the study model analysis. Analyses were performed at a 95% confidence interval (p = 0.05).

The validity and reliability analyses of the scales in the study were conducted through the SmartPLS program. It is preferable that factor‐loading values should be above 0.70, and indicator reliability values should be above 0.40. At the same time, Cronbach's alpha coefficient shows the model's reliability, and the combined reliability value of 0.70 and above shows that the scales in the study meet the necessary conditions. AVE (average variance extracted) values must be 0.50 and above for the model to have convergent validity (Ringle et al., 2020) (Table 1).

**TABLE 1:** Validity and reliability values of the measurement model.

Variables	Indicators	Outer loadings	Cronbach's alpha ≥0.70	Composite reliability (CR) ≥0.70	AVE ≥ 0.50
Workplace incivility	WI1	0.818	0.894	0.916	0.611
	WI2	0.712			
	WI3	0.826			
	WI4	0.783			
	WI5	0.838			
	WI6	0.748			
	WI7	0.740			
Psychological resilience	PR1	0.758	0.847	0.886	0.565
	PR2	0.757			
	PR3	0.726			
	PR4	0.769			
	PR5	0.712			
	PR6	0.788			
Work stress	WS1	0.782	0.894	0.919	0.653
	WS2	0.812			
	WS3	0.779			
	WS4	0.874			
	WS5	0.819			
	WS6	0.780			
Intention to quit	IQ1	0.843	0.841	0.904	0.759
	IQ2	0.895			
	IQ3	0.874			
Presenteism	P1	0.806	0.921	0.941	0.762
	P2	0.911			
	P3	0.906			
	P4	0.898			
	P5	0.838			

The criteria suggested by Fornell and Larcker (1981) were used to determine discriminant validity. According to the Fornell and Larcker (1981) criterion, the square root of the AVE values of the structures included in the research should be higher than the correlations between the structures included in the research (Table 2).

**TABLE 2:** The mean and discriminant validity results of the scales.

	WI	PR	WS	IQ	*P*	Mean	SD
Workplace incivility	0.782*					2.04	0.85
Psychological resilience	−0.321	0.752*				3.17	0.87
Work stress	0.498	−0.431	0.808*			3.18	0.98
Turnover intention	0.408	−0.311	0.479	0.871*		2.97	1.11
Presenteism	0.374	−0.424	0.477	0.272	0.873*	2.23	1.00
RMStheta: 0.121; SRMR: 0.057; Chi‐square: 718.848; NFI: 0.855							

*Note*: * = $\sqrt{\mathrm{AVE}}$.

Abbreviations: IQ, turnover intention; P, presenteism; PR, psychological resilience; WI, workplace incivility; WS, work stress.

### Model fit measures in PLS‐SEM

Standardized root mean square residual (SRMR) is the root mean square discrepancy between the observed and model‐implied correlations. Because the SRMR is an absolute measure of fit, a zero value indicates a perfect fit. A value less than 0.08 is generally considered a good fit (Hair et al., 2017).

Root mean square residual covariance (RMStheta) follows the same logic as SRMR but relies on covariances. RMStheta values below 0.12 indicate a well‐fitting model, whereas higher values indicate a lack of fit (Hair et al., 2017).

The non‐fuzzy index (NFI) was developed by Roubens (1978). Its formula incorporates the a posteriori probabilities of an observation belonging to a particular segment. This should not be confused with the normed fit index (also NFI) used in covariance‐based structural equation modelling (Garson, 2016).

### Ethical considerations

The study has been conducted according to the principles of the Declaration of Helsinki (2013). Before the study, we obtained the ethical approval of the ethics committee.

## RESULTS

### Nurses’ sociodemographic characteristics

Of the individuals participating in the study, 69.5% were female, and 30.5% were male. Regarding their educational status, 66.2% received undergraduate degrees, 19.2% associate degrees, 9.3% graduated, and 4.9% had high school education. The participants were between 18 and 53, with an average age of 31.05 ± 7.12. Their duration of average professional experience was 9.12 ± 7.66 years. On the other hand, 50.3% of the participants had a relaxed personality, whereas 48% had an anxious personality.

### Validity and reliability

Before the study model analysis, the validity and reliability analyses of the scales in the study were conducted through the SmartPLS program. The values obtained are shown in Table 1. According to Table 1, it has been observed that the values in the study are at an acceptable level. At the same time, a reliability value of 0.70 and above shows that the scales in the study meet the necessary conditions. The model has convergent validity (AVE ≥ 50). As a consequence of these findings, it can be said that the model is reliable and meets the convergent validity criteria.

### Mean and discriminant validity

In the study, Fornell–Larcker criteria and the values concerning the correlations among latent variables were examined for the model's validity. Table 2 shows the means of the scales and the discriminant validity results. According to the table, the cross‐values showing the square roots of AVE in the model were larger than the correlation values of the other latent variables in the rows and columns. Therefore, the model meets the discriminant validity criteria.

As seen in Table 2, the SRMR value of the model was found to be 0.05. This value conforms with the criterion of less than 0.08 in the literature. The NFI value of 0.855 indicates that the model is a well‐fitting model. The RMStheta value was 0.121. This value is greater than the reference value of 0.12. It has been determined that the model is well‐fitting within the framework of values other than the RMStheta value.

### Structural equation modelling

**FIGURE 1:**
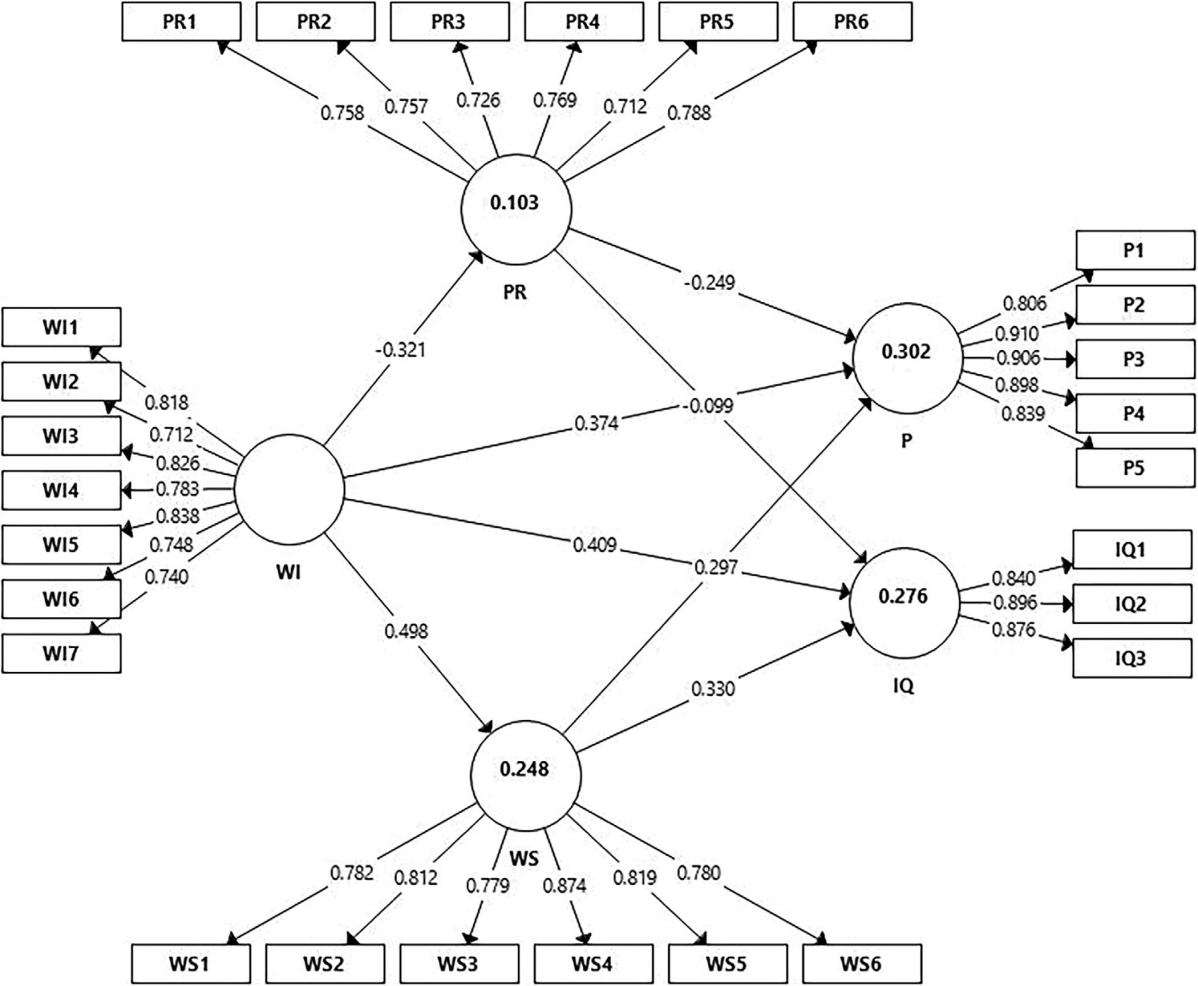
Model output.

The result of the structural model obtained after the analysis performed through the Smart PLS program in accordance with the study model is shown in Figure 1.

According to the results, while workplace incivility had a positive and significant effect on turnover intention (β = 0.409, t = 8.645, p < 0.001), presenteeism (β = 0.374, t = 6.719, p < 0.001), and work stress (β = 0.498, t = 12.028, p < 0.001), it had a negative and significant effect on psychological resilience (β = −0.321, t = 6.204, p < 0.001). Although psychological resilience had a negative and significant effect on presenteeism (β = −0.249, t = 4.916, p < 0.001), it had no significant effect on turnover intention (β = −0.099, t = 1.556, p = 0.120). It was determined that work stress positively and significantly affected turnover intention (β = 0.330, t = 5.072, p < 0.001) and presenteeism (β = 0.297, t = 5.467, p < 0.001). In addition, according to the findings related to indirect effects, it was found that psychological resilience had no mediating effect (β = 0.032, t = 1.458, p = 0.145) on the effect of workplace incivility on turnover intention. However, psychological resilience had a mediating effect (β = 0.080, t = 3.915, p < 0.001) on the effect of workplace incivility on presenteeism. It was found that work stress had a positive and significant effect on the effect of workplace incivility on turnover intention (β = 0.164, t = 4.451, p < 0.001) and presenteeism (β = 0.148, t = 4.898, p < 0.001). According to these findings, it is seen that hypotheses H_1_, H_2_, H_3_, H_4_, H_6_, H_7_, H_8_, H_10_, H_11_, H_12_ were accepted, but hypotheses H_5_ and H_9_ were rejected.

### DISCUSSION AND CONCLUSIONS

The study results are essential in revealing the factors affecting the nurses' turnover intention and presenteeism. According to OECD 2019 data, the percentage of nurses per 1,000 people in Turkey is 2.4 (OECD, 2021). This number is well below the OECD average of 8.8 (OECD, 2021). Mehmood et al. (2023) state that high workload and lack of staff bring high job stress and workplace incivility. According to the OECD (2021) report, there is a significant deficiency in the number of nurses in Turkey; therefore, working nurses must do their jobs efficiently and effectively. For nurses to work effectively and efficiently, it is important to understand and predict some factors, such as workplace incivility, intention to leave and presenteeism. The results of this study reveal that workplace incivility has a positive effect on presenteeism, turnover intention and work stress but a negative effect on psychological resilience. According to previous studies, the behaviours of incivility that nurses are exposed to in the workplace increase their turnover intention (Arslan Yürümezoğlu & Kocaman, 2019). Additionally, it is stated that there is a positive relationship between workplace incivility and presenteeism (Taylor et al., 2021) and work stress (Oyeleye et. al. et al., 2013). Due to the nature of the stressful environment of the nursing profession, it is natural that nurses' attitudes and behaviours towards work and the workplace are affected by the uncivil behaviours they encounter. The results obtained from this study and previous studies in the literature (Arslan Yürümezoğlu & Kocaman, 2019; Oyeleye et al., 2013; Taylor et al., 2021) conform to each other. Another result of this study shows a negative relationship between the psychological resilience of nurses and their perceptions of incivility in the workplace. This result shows that nurses with high psychological resilience can recover quickly from the effects of the events they encounter and are less affected by uncivil behaviours. Accordingly, they suffer relatively less from the consequences of incivility.

The results obtained from the study indicate that work stress has a positive effect on turnover intention and presenteeism. Findings exist in the literature showing that rising work stress increases turnover intention (Çetin Aydın et al., 2020) and presenteeism (Jiang et al., 2021). The results of this study indicate that work stress plays a mediating role in the positive effect of incivility in the workplace encountered by nurses on turnover intention and presenteeism. According to this, the nurses' perceived work stress further increases the effect of uncivil behaviours on their turnover intention and presenteeism. These results adversely affect the attitudes and behaviours of nurses related to their work due to the unfavourable conditions they are exposed to in the working environment (uncivil behaviour and work stress). Considering the importance of the services provided by nurses for human health and that health services do not have fault tolerance, it is likely that uncivil behaviours towards nurses adversely affect nurses, patients and the community.

The current study's results reveal that the behaviours of incivility encountered by nurses in the workplace increase their presenteeism and turnover intention, and work stress further strengthens these effects. Kanita and Naik (2021) stated that the experience of incivility causes stress among nurses, which may affect their intention to leave. Therefore, considering only workplace incivility is a critical administrative mistake. It is also essential to consider work stress, which will increase the potential effects of workplace incivility. Therefore, it is recommended that nursing and health managers first identify the stress factors in the workplace and be determined to fight them.

This study's other result indicates that although psychological resilience reduces presenteeism, it does not affect turnover intention. Another study's results show that resilience plays a mediating role in the effect of incivility encountered by nurses on presenteeism. According to these results, the psychological resilience of nurses will play an important role in reducing presenteeism. The psychological resilience of nurses is a factor that can help them eliminate their negative emotions and attitudes. Thus, organizing training and providing psychological support to increase nurses' psychological resilience may enable nurses to develop more positive feelings about their jobs and workplaces.

When the effects of workplace incivility on turnover intention and presenteeism are evaluated together with the critical nurse shortage in healthcare settings, the positivity of the work environment is extremely important. In this case, the main task falls to the administrators because they can reduce workplace incivility. Depending on organizational policies, employees may prefer to keep workplace incivility under control (Özdemir, 2023). Promoting courtesy in the workplace is essential to increase nurses' commitment to patients and the institution (Kanitha & Naik, 2021). Accordingly, workplace civility should be encouraged for the continuation of nurses' commitment to their profession and their institutions.

### Implications for nursing management

This study presents valuable results regarding the consequences of workplace incivility and stress, antecedents for nurses’ presenteeism and turnover intention. It is seen that many of the factors affecting presenteeism and turnover intention are related to the internal environment of the organization. Considering the potential consequences, the importance of combating presenteeism and turnover intention becomes evident. The study provides data to help nursing managers in this combat.

### Limitations

This study is a cross‐sectional study. Because the participants evaluated themselves in the questionnaires, they may have given biased responses. Another issue is that the participants exposed to incivility at work may have been more willing to participate in the survey. Another limitation is that the scales used in this study are intended to reveal only the main problem and are not suitable for in‐depth analyses of research topics. Since this study was conducted in only one hospital, its results can be generalized to the hospital where the research was conducted. In this study, only nurses were considered. All healthcare professionals can be included in future research. Thus, the existing problems in the health sector can be identified more deeply, and steps can be taken to produce more radical solutions.

## AUTHOR CONTRIBUTIONS

*Study design*: AD, ÖÜ, HT. *Data collection*: HT, YEÖ, ÖÜ. *Data analysis*: AD. *Study supervision*: ÖÜ. *Manuscript writing*: AD, HT, ÖÜ. *Critical revisions for important intellectual content*: ÖÜ, YEÖ.

## CONFLICT OF INTERESTS STATEMENT

The authors declare that there are no conflicts of interest.

## ETHICAL CONSIDERATIONS

The study has been conducted according to the principles of the Declaration of Helsinki (2013). Before the study, we obtained the ethical approval of the Ethics Committee of Sakarya University (approval dated 16 March 2021 and approval number E‐61923333‐050.99‐24074).
